# A Rare Case of Isolated Extrapulmonary Sarcoidosis With Renal Involvement Sans Pulmonary Findings: Diagnostic Challenges and Clinical Insights

**DOI:** 10.7759/cureus.67092

**Published:** 2024-08-17

**Authors:** Bipin Adhikari, Biplab Adhikari, Ashok kumar Kanugula, Prekshya Parajuli, Sonu Singh

**Affiliations:** 1 Internal Medicine, Wellstar Spalding Medical Center, Griffin, USA; 2 Internal Medicine, University of Louisville, Louisville, USA; 3 Internal Medicine, Essen Health Care, New York, USA; 4 Nephrology, Wellstar Spalding Medical Center, Griffin, USA

**Keywords:** renal stones, angiotensin-converting enzyme, sarcoid granulomas, calcinosis cutis, atypical presentation of sarcoidosis, chronic granulomatous disease, extrapulmonary manifestation of sarcoidosis, severe hypercalcemia, renal sarcoidosis

## Abstract

The cause of sarcoidosis is unknown, and it affects multiple systems with granulomas. Lung lesions are typical, but extrapulmonary findings, especially lymphadenopathy, are present in a significant number of cases. Isolated renal involvement is rare. The presence of noncaseating granulomas on biopsy is a hallmark of sarcoidosis.

We present the case of a 59-year-old male with recurrent renal stones who presented with renal failure. The initial diagnosis was challenging due to normal chest imaging and no pulmonary involvement. However, his delayed presentation of calcinosis cutis, an increase in angiotensin-converting enzyme (ACE) level, and the biopsy of the palm lesion with noncaseating granulomas helped us reach the diagnosis. He was started on prednisolone and achieved remission. The report also intends to show that patients with sarcoidosis can present without lung involvement, and physicians should consider sarcoidosis as their differential diagnosis for idiopathic hypercalcemia even if it has no lung or skin findings.

## Introduction

Sarcoidosis is a spectrum of disorders characterized by the presence of granulomas across various bodily systems. Histological detection of non-caseating granulomas (consisting of a tightly packed central area composed of macrophages, epithelioid cells, multinucleated giant cells, and T lymphocytes) is necessary for diagnosing sarcoidosis, although the exact cause of granulomas remains unknown [1]. Pulmonary involvement is seen in over 95% of patients [2]. Other common features consist of systemic symptoms such as fatigue, weight loss, and extrathoracic involvement like uveitis, thyroid dysfunction, erythema nodosum, osteoporosis, renal dysfunction, facial palsy, seizures, and hepatosplenomegaly [2,3]. Individuals affected by this condition may experience nephrocalcinosis, nephrolithiasis, interstitial nephritis, or even renal failure [2]. The diagnosis of sarcoidosis can be established using clinical, laboratory, and radiographic evidence, along with a tissue biopsy. It is crucial to rule out other causes of granulomatous diseases, such as mycobacterial infections [4].

Renal involvement in sarcoidosis is quite uncommon as an extrapulmonary manifestation and may present as hypercalcemia, tubular or glomerular dysfunction, and/or granulomatous interstitial nephritis [5]. Most sarcoidosis patients will go into remission without needing specific treatment. However, around one-third will suffer from chronic, potentially severe disease, and the specific mortality rate can reach up to 5% [6]. Treatment primarily involves the use of corticosteroids or immunosuppressive agents to manage symptoms [7].

All patients diagnosed with sarcoidosis should be assessed for renal involvement to prevent significant chronic kidney disease. In cases of hypercalcemia or hypercalciuria, levels of 25-hydroxyvitamin D3, 1,25-dihydroxyvitamin D, and parathyroid hormone should be measured to determine the extent and cause of calcium dysregulation. The case presented highlights the atypical presentation of sarcoidosis with isolated renal involvement and provides insights into its diagnostic challenges and the importance of an approach focused on the recurrent finding of hypercalcemia of unknown origin.

## Case presentation

**Figure 1:**
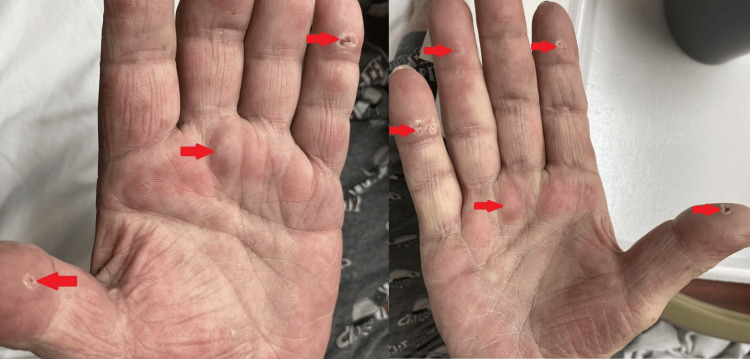
Photographs of the patient’s hands showing the hardening of the palmar surface of bilateral hands due to calcinosis cutis (red arrows)

A 59-year-old Caucasian male, presented with upper abdominal pain for five days prior to presentation. The dull, aching pain was non-radiating in nature and associated with bilateral lower limb swelling with no specific aggravating or relieving factors. He does have a history of hypertension, extensive arteriosclerosis, recurrent kidney stones with four past lithotripsies, and recurrent hypercalcemia. He later got diagnosed with stage 3b chronic kidney disease which was believed to be due to recurrent hypercalcemia and nephrolithiasis. As part of his work history, the patient mentioned that he stays at home and occasionally participates in gardening activities. A review of the rest of the system was insignificant except as mentioned above. He was on calcitonin and zoledronic acid prescription for his raised calcium level. On examination, his vital signs were stable with bilateral pitting edema noted over his lower limbs. Dermatological findings revealed calcinosis cutis over his bilateral palmar lesion (Figure 1). No abnormalities were detected on examination of other bodily systems.

The initial laboratory findings revealed a raised white blood cell count with elevated blood urea nitrogen and creatinine levels. His estimated glomerular filtration rate (eGFR) was calculated to be 32 ml/min/1.73 m^2^. The serum calcium level was high, with a borderline low albumin level. The calculated corrected calcium level was in the higher range with a low serum parathyroid hormone level (Table 1).

**Table 1:** Laboratory tests of the patient

Test	Result	Reference range
White blood cells (WBC)	13,000	4,000-10,000
Blood urea nitrogen (BUN)	37 mg/dL	8-23 mg/dl
Creatinine	2.28 mg/dL	0.6-1.2 mg/dl
eGFR	32 ml/min/1.73 m^2^	>90 ml/min/1.73 m^2^
Calcium	14.9 mg/dL	8.5-10.2 mg/dl
Albumin	3.3 g/dL	3.5-5 g/dl
Corrected calcium	15.5 mg/dL	8.5-10.2 mg/dl
Parathyroid hormone (PTH)	4 pg/dL	14-65 pg/dl
Parathyroid hormone-related protein (PTHrp)	9 pg/mL	11-20 pg/ml
Angiotensin-converting enzyme (ACE)	138 micrograms/L	<40 micrograms/L
1,25-dihydroxyvitamin D	126 ng/mL	18-73 ng/ml

**Figure 2:**
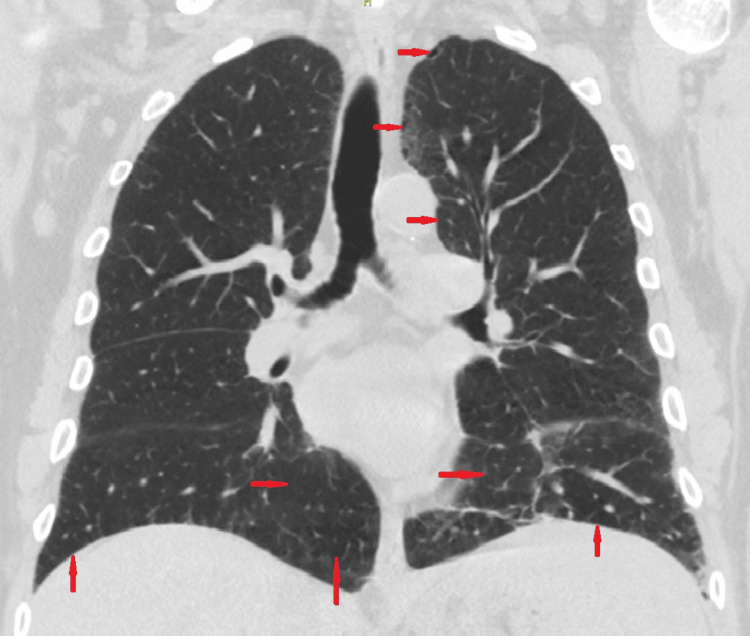
CT scan of the chest (coronal view) The red arrows indicate emphysematous changes seen in the lungs.

A chest CT scan displayed some emphysematous changes (Figure 2), with normal results seen on the CT abdomen and pelvis. Parathyroid hormone-related protein level was low (Table 1), with no paraprotein detected on serum and urine protein electrophoresis. His serum erythrocyte sedimentation rate (ESR), C-reactive protein (CRP), and thyroid function tests were all within the normal range. Autoimmune disease and malignancy were not present.

**Figure 3:**
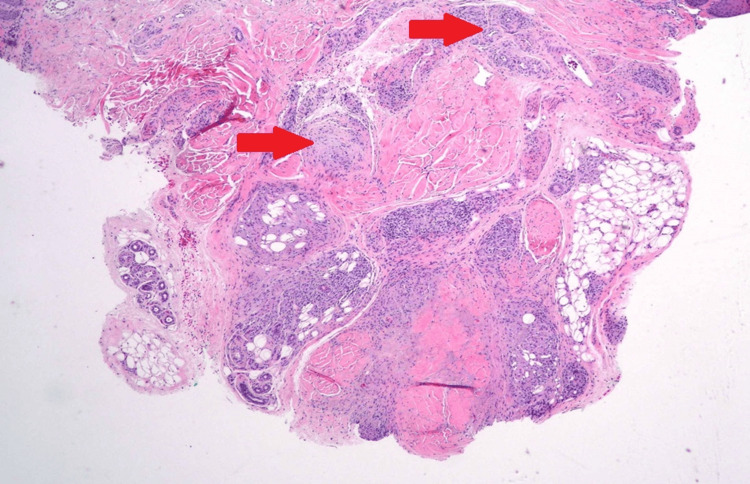
Biopsy of the skin lesion revealing sarcoid granulomas (red arrows)

The levels of angiotensin-converting enzyme (ACE) and 1,25-dihydroxyvitamin D were measured, and the results revealed elevated levels in both (Table 1). Later, a skin biopsy was performed, and it showed non-caseating granuloma (Figure 3), confirming a diagnosis of sarcoidosis.

Following a diagnosis of sarcoidosis, low-dose prednisolone (20 mg per day) was started under careful monitoring, as the patient had chronic kidney disease and was also continuing calcitonin and zoledronic acid. As his calcium levels were in the normal range on regular follow-ups (Table 2), his calcitonin and zoledronic acid were stopped. The patient will continue to receive regular follow-ups to see if he develops any complications or signs of adverse drug reactions due to steroids.

**Table 2:** Laboratory tests of the patient on the follow-up visit after a month eGFR: Estimated glomerular filtration rate

Test	Result	Reference range
Sodium (Na)	136	135-145 mEq/L
Potassium (K)	4.7	3.7-5.2 mEq/L
Blood urea nitrogen (BUN)	28	8-23 mg/dl
Creatinine	1.07	0.6-1.2 mg/dl
eGFR	80	>90 ml/min/1.73 m^2^
Calcium	8.9	8.5-10.2 mg/dl

## Discussion

Sarcoidosis can impact different body systems and requires prompt identification and treatment. A common imaging finding in the typical diagnosis of sarcoidosis is asymptomatic lymphadenopathy, often involving bilateral hilar lymphadenopathy. Nevertheless, pulmonary infiltrates might be absent. In 25% to 30% of cases, these typical features may not be seen [8]. Angiotensin-converting enzyme level demonstrates significant diagnostic potential in sarcoidosis, as shown in this patient. Elevated ACE levels are a key diagnostic indicator due to their sensitivity and specificity, which are 70% and 79%, respectively [9].

Up to 0.7% of individuals with sarcoidosis can have renal involvement [2]. Microscopic renal involvement may exist, but noticeable renal involvement is uncommon [10]. The clinical characteristics are commonly caused by conditions secondary to nephrolithiasis, nephrocalcinosis, granulomatous interstitial nephritis, glomerular disease, and tubular dysfunction [6]. Chronic renal failure can occur in sarcoidosis, with nephrocalcinosis being the primary reason for this condition [11].

This patient had multiple visits for renal stones, and his calcium was elevated. Nevertheless, sarcoidosis was not initially believed to be a potential diagnosis due to the absence of hilar lymphadenopathy on the chest CT scan, and isolated renal involvement on its own is uncommon. While being monitored regularly, the patient developed cutaneous calcinosis as well as increased levels of ACE and vitamin D. A biopsy of the palm lesion revealed a non-caseating sarcoidal granuloma, confirming the diagnosis of sarcoidosis. He was given corticosteroids, which work as anti-inflammatory agents in sarcoidosis and inhibit the activity of 1-α-hydroxylase in macrophages. They also reduce the absorption of calcium in the intestines and prevent bone breakdown by osteoclasts. Hypercalcemia is an indication to start treating patients with sarcoidosis with steroids, even if their symptoms are mild [12]. 

## Conclusions

This case highlights the atypical features of sarcoidosis and the diagnostic challenges they pose. Sarcoidosis is typically recognized as a disease affecting the lungs or lymph nodes, with renal involvement being uncommon. In our case, the patient experienced recurrent episodes of hypercalcemia without other typical manifestations of sarcoidosis, leading to diagnostic difficulty. A biopsy of calcinosis cutis revealed noncaseating granulomas, confirming the diagnosis of sarcoidosis with atypical renal involvement. Management was initiated with prednisolone therapy to reduce inflammation. This case emphasizes the importance of considering biopsy in patients presenting with recurrent hypercalcemia and without any pulmonary or lymph node findings to effectively diagnose sarcoidosis and implement appropriate therapeutic strategies to prevent complications.
